# Prince of Wales's Hospital Fund

**Published:** 1898-01-01

**Authors:** 


### PRINCE OF WALES'S HOSPITAL FUND.

### A GREAT ROLL OF BRITISH CHILDREN.—A NEW CHRISTMAS GAME.

The new Christmas game for children, consisting of the purchase by parents or children of a stamp album of British manufacture, fixing therein the two Prince of Wales's Hospital Fund Stamps, and then presenting the children to be photographed free of cost by Messrs. Speaight, 178, Regent-street, is becoming very popular. Messrs. Speaight have opened special studios, where the children's photographs will be taken free of cost, and where stamp albums and stamps can be obtained by parents and children. Owing to the large numbers wishing to be photographed, two days' notice should be sent to Messrs. Speaight, and the stamps and albums should be obtained at once from them, or from the nearest bookstall, bookseller, or chemist. With the Prince's approval it has been determined to create a great roll of children subscribers, and a midget photograph and the autograph of each child who purchases the album and places in it a half-crown and shilling stamp will be entered in the great roll, which will be offered for the acceptance of H.R.H. the Prince of Wales. H.R.H. the Duke of York has this week commanded Messrs. Speaight to Sandringham to take a special photograph of his children, so that they may appear in the roll as the first children subscribers. It is desirable that the most beautiful children should appear in this roll, a fact of which every mother of beautiful children should make a note, as the number of children to appear in it must necessarily be limited strictly to 100,000, and an early appointment should be made with Messrs. Speaight if disappointment is to be avoided. Many a hospital sister may like to have one or more children in her ward photographed for the Great Roll of Ministering Children.

### COMING OF AGE IN 1897.

Messrs. Martin and Sallnow, 416, Strand, W.C., have agreed to open a second roll of adult subscribers, being the members of both sexes who attain their majority in the Diamond Jubilee year 1897. The Registrar-General states that upwards of 90,000 persons have attained their majority in London alone during 1897. It is proposed to offer this roll also for the acceptance of the Prince of Wales, and Messrs. Martin and Sallnow have agreed to take a midget photograph of everyone so attaining their majority who purchases a stamp album and fixes in it the two stamps issued in support of the Prince of Wales's Hospital Fund for London. The importance of these proposals consists in the fact that each stamp album getting into the proper hands creates an annual subscriber to the hospitals.

